# The Prevalence of Disabilities Among Vietnamese-Origin Older Adults in the United States

**DOI:** 10.1177/30495334261418950

**Published:** 2026-02-18

**Authors:** Hoang T. Nguyen, Christina E. Miyawaki, Kyriakos S. Markides

**Affiliations:** 1 University of Texas Medical Branch, Galveston, TX, USA; 2 University of Houston Graduate College of Social Work, Houston, TX, USA

**Keywords:** aging, disability, quality of life, race/ethnicity, observational study

## Abstract

**Background:**

Little information is available on the disability status of the diverse Asian-origin older population in the United States (U.S.). Limited literature has shown that Vietnamese-origin older adults have the highest prevalence of disability among all Asian-origin groups. Our primary objective was to illustrate how Vietnamese-origin older adults differed from those of other Asian and non-Hispanic White-origin.

**Methods:**

We updated a previous report and computed national estimates of the prevalence of different types of disability for various ethnic populations using data from the 2017 to 2021 American Community Survey. Survey weights and the jackknife method were used for population estimates.

**Results:**

Results showed that all Asian-origin older adults as a group reported higher prevalence of disability in all categories (independent living, self-care, cognitive, ambulatory, and vision) except for hearing disability, compared to non-Hispanic Whites. Among the Asian-origin groups, Vietnamese-origin older adults of both genders reported the highest prevalence of disability, which was consistent with previously reported results in 2011.

**Conclusions:**

Owing to their refugee and socioeconomic statuses and other factors, Vietnamese-origin older adults in the U.S. are an especially vulnerable population whose poor overall health and health care needs deserve the attention of both health and social policy makers.

## Introduction

A large influx of Vietnamese refugees and immigrants arrived in the United States in three waves: the first, immediately at the end of the Vietnam War in 1975; the second, beginning a few years later from 1979 to the early 1980s, often referred to as “boat people” (Klineberg & Wu, 2023; Rkasnuam & Batalova, 2014); and the third, starting around 1985 and lasting throughout the 1990s through the United Nations’ Orderly Departure Program. By 2022, the U.S. was home to an estimated 2.3 million people of Vietnamese-origin, of whom over a quarter million were 65 years and older (Rico et al., 2023).

Only three studies, over the years, have computed national disability prevalence rates for older adults of Asian-origin (Fuller-Thomson et al., 2011; Mutchler et al., 2007; Yang et al., 2012) with other studies relying on data from state (New York and California) and local (Houston) surveys (Miyawaki et al., 2022, 2024, 2025; P. Mui et al., 2017; A. C. Mui & Shibusawa, 2008). These studies have consistently painted a poorer disability profile for older adults of Vietnamese-origin, with respect to mobility restrictions, cognitive difficulties, and independent living, compared to non-Hispanic White, as well as to other Asian-origin populations. Migrant selectivity effects do not necessarily apply to Vietnamese-origin older adults, who are predominantly refugees (Fuller-Thomson et al., 2011). Below, we intend to replicate Fuller-Thomson et al.’s (2011) study and report updated national estimates of disability using data for 2017 to 2021 from the American Community Survey (ACS). We report the prevalence of disabilities for the six largest non-Hispanic Asian-origin older adults (≥65 years old) in the U.S.: Vietnamese, Chinese, Filipino, Japanese, Korean, and Asian Indian compared to non-Hispanic Whites. We also estimate the risk of disability for each Asian-origin subgroup relative to non-Hispanic Whites using unadjusted and age-adjusted odds ratios. Many studies have used all Asian subgroups as one aggregate group, missing variability among Asian populations. Thus, the strength of this study is to examine the variability of subgroup differences using the updated dataset. We pay special attention to older Vietnamese adults, given their previously shown poor disability rates.

## Methods

### Data and Sample

We employed data from the 2017 to 2021 Public Use Microdata Sample (PUMS) from the ACS. The ACS is a continuous rolling survey administered by the U.S. Census Bureau to a random sample of approximately 295,000 household addresses a month across the U.S. The survey asks one person/household to complete a standardized questionnaire to obtain data for each member of the household. The data for the 5-year file were based on a sample of approximately 5% of the U.S. population.

We restricted the sample to adults 65 years or older living in a household setting. People living in group quarters, such as nursing homes, were excluded. In addition, the sample includes only individuals who reported being of single ethnic-origin, not Hispanic, and belonging to one of the above six major Asian-origin groups, which represent 85% of the U.S. Asian-origin population. Individuals who reported being White of non-Hispanic origin represent the primary comparison group. Individuals who reported being of more than one Asian-origin group or mixed race were excluded to prevent multiple group assignments and double-counting.

### Measurements

#### Census Disability Measures

The ACS contains six questions related to disability. Respondents answer yes or no to these six questions for each member of the household. The severity level for each disability is not assessed. The six questions are:

1. Is this person deaf or does he/she have serious difficulty hearing?
2. Is this person blind or does he/she have serious difficulty seeing even when wearing glasses?
3. Because of a physical, mental, or emotional condition, does this person have serious difficulty concentrating, remembering, or making decisions?
4. Does this person have serious difficulty walking or climbing stairs?
5. Does this person have difficulty dressing or bathing?
6. Because of physical, mental, or emotional condition, does this person have difficulty doing errands alone such as visiting a doctor’s office or shopping?

These six questions correspond to disability in hearing, vision, cognition, ambulation, self-care, and independent living, respectively.

#### Race/Ethnicity

Survey respondents report their racial and ethnic group for themselves and others in the same household. We first excluded all Hispanics from the sample, then coded the race/ethnic groups as follows: White-alone, Vietnamese-alone, Chinese-alone, Korean-alone, Japanese-alone, Filipino-alone, and Asian Indian-alone. The ACS denotes “alone” to distinguish from people who identified as being of two or more races/ethnicities.

#### Age, Sex, Marital Status, and Education Level

The ACS reports a person’s age in years (top-coded at 99 years old). We limit our analysis to individuals aged 65 years or older. Sex is reported as either male or female. Marital status is a five-category variable: married, separated, divorced, widowed, or never married. Education level is defined as the highest grade level or highest degree a person has achieved in the U.S. or elsewhere and is grouped into five categories: (1) No formal schooling, (2) Eighth grade or less (first to eighth grade), (3) Some high school (grade 9–12, but did not graduate), (4) High school graduate (diploma or equivalent), and (5) College graduate (associate degree, bachelor degree, master degree, professional degree, doctorate degree).

#### Poverty Level

The income-to-poverty ratio is a variable computed by the ACS. This variable shows where a person/household is in relation to the poverty threshold. Poverty thresholds are set dollar amounts based on family size, number of children, and whether the householder is 65 years or older. We grouped poverty level into four categories: 100% or below, 101% to 150%, 151% to 200%, and 201% or higher.

#### Year and Age of Entry

The year of entry is asked for all persons not born in the U.S. and denotes the latest year a person came to live in the U.S. age of entry is a computed variable that subtracts the year of entry from the survey year for those who were immigrants.

#### People in Household

Respondents are asked to enumerate all persons residing at the present address whether or not they are related to each other. The number of people in the household reflects this enumeration.

#### English Fluency

Respondents are instructed to answer yes if they sometimes or always spoke a language other than English at home. Slang and a few expressions do not count as speaking another language. If a person speaks a language other than English at home, then a follow-up question asks “How well does the person speak English?” with four response choices: “Very Well,” “Well,” “Not Well,” and “Not at All.”

### Analytical Methods

All analyses incorporated the respondents’ survey weights for the point estimate of the U.S. population. The standard errors of the point estimate were calculated using the jackknife method. Eighty replicate weights and a jackknife coefficient of 4/80 provided by ACS were used to remove survey bias and to obtain 95% confidence intervals. Analyses were restricted to the sample of racial/ethnic groups defined in the study. In addition, analyses were repeated separately for men and women. Logistic regression models were used to compare differences in odds of having a disability between the non-Hispanic White group and each Asian-origin group. Odds ratios and 95% confidence intervals were computed in the unadjusted and age-adjusted models. SAS Software 9.4 and SAS/STAT 15.1 were used to analyze the data.

## Results

### Demographic Differences of Sample Populations

Table 1 shows the population characteristics of older adults by race/ethnicity. All racial and ethnic groups except Japanese had the highest proportion at ages of 65 and 74, whereas Japanese had the highest proportion in the 80 years and older category. In all groups, the majority of the subjects were married, followed by the widowed group. With respect to educational status, subjects of Vietnamese origin were less educated than all the other groups. With respect to poverty level, the Vietnamese, Chinese, and Korean groups had the highest proportion living below the poverty level.

**Table 1.** Population Characteristics of Persons Aged 65 and Older (ACS 2017–2021).

	White	Vietnamese	Chinese	Filipino	Japanese	Korean	Asian Indian
Sample size	2,412,999	11,737	32,388	24,625	12,125	10,231	16,759
Population estimate	38,933,254	242,743	621,461	472,928	218,058	210,312	356,525
Age group	Percent	Percent	Percent	Percent	Percent	Percent	Percent
65–69 years old	32.6	38.4	35.8	35.7	27.7	31.3	36.7
70–74	27.0	25.2	25.0	27.3	22.5	26.5	28.0
75–79	17.5	16.8	14.9	17.3	15.4	20.4	19.3
80 or older	22.9	19.6	24.3	19.7	34.4	21.8	16.0
Sex							
Male	45.9	45.6	45.0	37.5	37.8	37.6	51.3
Female	54.1	54.4	55.0	62.5	62.2	62.4	48.7
Marital status							
Married	59.8	62.4	67.9	61.5	55.7	65.4	72.1
Widowed	20.8	21.1	19.8	23.2	24.4	20.6	21.4
Divorced	14.0	8.6	7.3	8.4	11.3	9.8	4.1
Separated	0.7	2.1	1.3	1.8	0.7	1.6	0.7
Never married	4.7	5.9	3.8	5.1	7.9	2.6	1.8
Education level							
No formal schooling	0.7	17.6	11.0	2.8	1.3	5.2	7.2
8th grade or less	2.3	11.1	12.1	6.3	1.8	7.9	5.9
Some H.S.	5.5	14.0	9.3	4.5	4.5	8.2	9.0
H.S. graduate	52.2	35.1	26.2	32.2	43.1	38.6	20.6
College graduate	39.4	22.2	41.5	54.2	49.3	40.1	57.3
Poverty level							
100% or below	7.3	16.5	17.9	7.3	6.9	18.2	7.3
101–150%	7.6	13.5	10.3	5.7	5.4	12.0	5.0
151%–200%	8.6	10.0	6.8	6.0	6.2	9.2	5.4
201% or higher	76.4	60.0	65.0	81.0	81.4	60.6	82.4
Year of entry							
Before 1975	3.4	9.0	22.2	29.2	22.3	29.0	23.1
1975–1,999	1.6	72.6	46.4	46.4	13.3	58.1	48.6
2000–2021	0.6	15.9	21.5	18.3	2.4	9.7	25.9
Born in the U.S.	94.3	2.5	9.9	6.1	62.0	3.2	2.4
Age of entry							
0–4	0.6	0.1	0.5	0.7	1.0	0.2	0.3
5–12	0.6	0.3	1.5	1.1	1.5	0.4	0.3
13–20	0.9	2.6	6.1	5.8	3.3	4.0	3.1
21–39	2.2	40.2	36.4	47.1	24.8	58.4	46.5
40–64	1.1	48.0	34.0	32.0	6.0	29.7	34.2
65+	0.3	6.3	11.6	7.3	1.3	4.1	13.2
Born in the U.S.	94.3	2.5	9.9	6.1	62.0	3.2	2.4
People in household							
Single person	27.5	9.6	14.8	8.9	26.1	19.3	6.2
Two people	57.9	31.2	43.6	34.5	49.4	51.2	36.4
Three or more	14.5	59.2	41.6	56.6	24.5	29.6	57.3
Speaks English							
Very well	60.5	12.5	20.2	55.6	43.0	18.3	49.6
Well	19.9	24.6	21.8	30.4	36.9	31.8	23.1
Not well	14.8	43.9	30.8	12.7	18.2	39.2	17.3
Not at all	4.8	19.0	27.2	1.3	1.9	10.6	10.0

The vast majority of Vietnamese-origin subjects entered the United States at ages 21 to 64 (88.2%), much like the other Asian-origin groups (Chinese = 70.4%; Filipino = 79.1%; Korean = 88.1%; Asian Indian = 80.7%) except for the Japanese (30.8%), the majority of whom were U.S.-born. Also, the majority of the Vietnamese (59.2%), along with the Filipino (56.6%) and Asian Indian-origin subjects (57.3%), reported living in households of three or more persons, whereas only 14.8% of non-Hispanic Whites lived in households of three or more. Finally, the majority of the Vietnamese (62.9%) as well as Chinese-origin subjects (58%) reported not speaking English well or not at all.

### Prevalence and Odds of Disability

Table 2 presents the prevalence and odds of disability by race/ethnicity. Vietnamese-origin older adults had the highest prevalence of all disabilities except hearing. This pattern was reflected in the higher unadjusted odds ratios (OR) for independent living difficulty (*OR* = 1.78, *p* < .0001), self-care difficulty (*OR* = 1.75, *p* < .0001), cognitive difficulty (*OR* = 1.84, *p* < .0001), ambulatory difficulty (*OR* = 1.10, *p* < .0001), and vision difficulty (*OR* = 1.20, *p* < .0001) in comparison with non-Hispanic Whites. After adjusting for age the odds of independent living difficulty, self-care difficulty, and cognitive difficulty were two times higher than Whites (*OR* = 2.17, 2.03, and 2.07, *p* < .0001, respectively). Their ambulatory difficulty and vision difficulty were 21% and 30% higher than Whites, respectively.

**Table 2.** All Adults Aged 65 and Older: Population Prevalence and Risk of Disability by Race/Ethnicity.

Race/ethnicity	Independent living	Self-care difficulty	Cognitive difficulty	Ambulatory difficulty	Vision difficulty	Hearing difficulty
Population prevalence						
	Percent [95% CI]	Percent [95% CI]	Percent [95% CI]	Percent [95% CI]	Percent [95% CI]	Percent [95% CI]
White	12.2 [12.1, 13.3]	6.5 [6.40, 6.51]	7.2 [7.13, 7.29]	19.7 [19.6, 19.8]	5.52 [5.46, 5.58]	14.6 [14.6, 14.7]
Vietnamese	**19.8** [18.7, 20.9]	**10.8** [9.91, 11.6]	**12.5** [11.5, 13.4]	**21.3** [20.1, 22.4]	**6.54** [5.93, 7.14]	**11.8** [11.1, 12.6]
Chinese	**15.5** [15.0, 16.1]	**7.9** [7.50, 8.32]	7.7 [7.26, 8.04]	**16.1** [15.7, 16.6]	**4.62** [4.27, 4.97]	**9.5** [9.13, 9.93]
Filipino	**16.4** [15.7, 17.0]	**7.5** [7.03, 7.97]	**8.6** [8.05, 9.14]	20.0 [19.3, 20.7]	5.22 [4.78, 5.65]	**11.2** [10.7, 11.7]
Japanese	**14.4** [13.5, 15.3]	6.7 [6.11, 7.23]	**8.7** [8.09, 9.31]	**16.9** [15.9, 17.8]	**4.24** [3.71, 4.76]	**13.1** [12.4, 13.8]
Korean	12.1 [11.3, 13.0]	6.4 [5.67, 7.04]	6.7 [6.12, 7.36]	**14.4** [13.5, 15.4]	**3.82** [3.31, 4.32]	**8.3** [7.54, 8.96]
Asian Indian	**15.2** [14.4, 15.9]	**7.6** [7.04, 8.10]	**6.4** [6.00, 6.86]	**17.9** [17.1, 18.7]	**4.35** [4.01, 4.69]	**8.6** [8.05, 9.22]
Unadjusted Odds Ratios (O.R.)						
	O.R. [95% CI]	O.R. [95% CI]	O.R. [95% CI]	O.R. [95% CI]	O.R. [95% CI]	O.R. [95% CI]
Vietnamese	**1.78** [1.65, 1.91]	**1.75** [1.60, 1.92]	**1.84** [1.68, 2.00]	**1.10** [1.03, 1.18]	**1.20** [1.09, 1.32]	**0.78** [0.73, 0.84]
Chinese	**1.33** [1.27, 1.38]	**1.24** [1.17, 1.32]	**1.07** [1.01, 1.13]	**0.78** [0.76, 0.81]	**0.83** [0.77, 0.90]	**0.61** [0.59, 0.64]
Filipino	**1.41** [1.35, 1.48]	**1.18** [1.10, 1.26]	**1.21** [1.13, 1.30]	1.02 [0.98, 1.06]	0.94 [0.86, 1.03]	**0.73** [0.70, 0.77]
Japanese	**1.21** [1.13, 1.31]	1.04 [0.95, 1.13]	**1.23** [1.14, 1.32]	**0.83** [0.77, 0.89]	**0.76** [0.66, 0.86]	**0.88** [0.82, 0.94]
Korean	0.99 [0.92, 1.08]	0.98 [0.88, 1.10]	0.93 [0.84, 1.02]	**0.69** [0.64, 0.74]	**0.68** [0.59, 0.78]	**0.52** [0.48, 0.58]
Asian Indian	**1.29** [1.21, 1.36]	**1.19** [1.10, 1.28]	**0.88** [0.82, 0.95]	**0.89** [0.85, 0.94]	**0.78** [0.72, 0.85]	**0.55** [0.51, 0.60]
Age-adjusted Odds Ratios (O.R.)						
	O.R. [95% CI]	O.R. [95% CI]	O.R. [95% CI]	O.R. [95% CI]	O.R. [95% CI]	O.R. [95% CI]
Vietnamese	**2.17** [2.02, 2.35]	**2.03** [1.85, 2.22]	**2.07** [1.89, 2.26]	**1.21** [1.23, 1.30]	**1.30** [1.18, 1.43]	**0.84** [0.78, 0.90]
Chinese	**1.36** [1.30, 1.42]	**1.24** [1.17, 1.31]	**1.06** [1.00, 1.12]	**0.77** [0.74, 0.80]	**0.82** [0.75, 0.88]	**0.59** [0.56, 0.62]
Filipino	**1.65** [1.57, 1.72]	**1.31** [1.22, 1.40]	**1.32** [1.23, 1.41]	**1.01** [1.05, 1.14]	1.00 [0.92, 1.10]	**0.77** [0.74, 0.82]
Japanese	**0.88** [0.83, 0.95]	**0.77** [0.70, 0.84]	0.97 [0.90, 1.04]	**0.65** [0.61, 0.69]	**0.61** [0.53, 0.69]	**0.69** [0.65, 0.74]
Korean	1.04 [0.95, 1.14]	1.03 [0.91, 1.15]	0.96 [0.87, 1.05]	**0.69** [0.63, 0.74]	**0.69** [0.60, 0.79]	**0.52** [0.47, 0.57]
Asian Indian	**1.63** [1.54, 1.73]	**1.44** [1.34, 1.56]	1.02 [0.94, 1.10]	1.00 [0.95, 1.06]	**0.88** [0.81, 0.95]	**0.62** [0.57, 0.66]

*Note.* Reference group is non-Hispanic White. Bold font indicates statistical significance at *p* < .05 for logistic regression models.

Other primarily immigrant Asian-origin subgroups had disability patterns similar to the Vietnamese in comparison with non-Hispanic Whites for independent living, self-care, and cognitive difficulty (Chinese, Filipino, Korean, and Asian Indian). Despite their relatively older age, Japanese older adults were the healthiest population group compared to non-Hispanic Whites, as well as other Asian-origin immigrants. Non-Hispanic Whites reported significantly more hearing difficulty than each of the Asian-origin groups, as seen in both prevalence rates and odds ratios.

Tables 3 and 4 present results separately for men and women. Both Vietnamese men and women had the highest prevalence of all disabilities, except for hearing difficulty, compared to non-Hispanic Whites and other Asian-origin groups. Vietnamese women reported more difficulty than Vietnamese men for independent living, cognitive function, and ambulatory function. Similar patterns by gender are also evident among non-Hispanic Whites as well as all the other Asian-origin groups.

**Table 3.** Men Aged 65 and Older: Population Prevalence and Risk of Disability by Race/Ethnicity.

Race/ethnicity	Independent living	Self-care difficulty	Cognitive difficulty	Ambulatory difficulty	Vision difficulty	Hearing difficulty
Population prevalence						
	Percent [95% CI]	Percent [95% CI]	Percent [95% CI]	Percent [95% CI]	Percent [95% CI]	Percent [95% CI]
White	9.5 [9.45, 9.61]	5.57 [5.51, 5.64]	6.7 [6.64, 6.82]	17.2 [17.0, 17.3]	5.16 [5.09, 5.24]	18.8 [18.7, 18.9]
Vietnamese	**15.5** [14.1, 16.9]	**8.87** [7.74, 10.0]	**10.6** [9.51, 11.6]	17.2 [15.9, 18.5]	**6.31** [5.46, 7.16]	**13.5** [12.3, 14.6]
Chinese	**11.8** [11.2, 12.5]	6.14 [5.58, 6.71]	6.5 [5.93, 7.09]	**13.1** [12.5, 13.7]	**4.01** [3.59, 4.44]	**11.1** [10.5, 11.7]
Filipino	**12.9** [12.1, 13.7]	**6.49** [5.94, 7.05]	**7.7** [6.99, 8.32]	16.6 [15.7, 17.5]	5.39 [4.69, 6.09]	**14.0** [13.1, 15.0]
Japanese	9.0 [7.85, 10.2]	**4.31** [3.53, 5.09]	**5.5** [4.69, 6.33]	**13.1** [11.7, 14.6]	**3.59** [2.86, 4.31]	**16.1** [14.8, 17.5]
Korean	8.8 [7.66, 10.0]	5.14 [4.21, 6.06]	**5.2** [4.23, 6.26]	**10.7** [9.41, 12.0]	**2.69** [1.99, 3.40]	**9.1** [7.84, 10.3]
Asian Indian	**11.5** [10.7, 12.3]	**6.36** [5.71, 7.01]	**5.4** [4.86, 6.01]	**13.6** [12.8, 14.4]	**3.94** [3.38, 4.51]	**8.95** [8.11, 9.80]
Unadjusted Odds Ratios (O.R.)						
	O.R. [95% CI]	O.R. [95% CI]	O.R. [95% CI]	O.R. [95% CI]	O.R. [95% CI]	O.R. [95% CI]
Vietnamese	**1.74** [1.56, 1.93]	**1.65** [1.43, 1.90]	**1.64** [1.46, 1.83]	1.00 [0.91, 1.10]	**1.24** [1.07, 1.43]	**0.67** [0.61, 0.74]
Chinese	**1.28** [1.19, 1.36]	**1.11** [1.01, 1.22]	0.97 [0.88, 1.06]	**0.73** [0.69, 0.77]	**0.77** [0.69, 0.86]	**0.54** [0.51, 0.58]
Filipino	**1.40** [1.31, 1.50]	**1.18** [1.07, 1.29]	**1.15** [1.04, 1.26]	0.96 [0.90, 1.03]	1.05 [0.91, 1.21]	**0.71** [0.65, 0.76]
Japanese	0.94 [0.82, 1.08]	**0.76** [0.63, 0.92]	**0.81** [0.69, 0.95]	**0.73** [0.65, 0.83]	**0.68** [0.55, 0.85]	**0.83** [0.75, 0.92]
Korean	0.92 [0.79, 1.06]	0.92 [0.76, 1.11]	**0.77** [0.63, 0.94]	**0.58** [0.51, 0.67]	**0.51** [0.39, 0.67]	**0.43** [0.37, 0.50]
Asian Indian	**1.23** [1.14, 1.34]	**1.15** [1.03, 1.28]	**0.80** [0.71, 0.89]	**0.76** [0.71, 0.81]	**0.76** [0.65, 0.88]	**0.43** [0.38, 0.47]
Age-adjusted Odds Ratios (O.R.)						
	O.R. [95% CI]	O.R. [95% CI]	O.R. [95% CI]	O.R. [95% CI]	O.R. [95% CI]	O.R. [95% CI]
Vietnamese	**1.89** [1.69, 2.10]	**1.73** [1.51, 1.99]	**1.70** [1.51, 1.91]	1.03 [0.94, 1.13]	**1.27** [1.10, 1.46]	**0.68** [0.61, 0.75]
Chinese	**1.23** [1.15, 1.32]	1.06 [0.96, 1.17]	0.93 [0.85, 1.02]	**0.69** [0.65, 0.73]	**0.74** [0.66, 0.82]	**0.50** [0.47, 0.54]
Filipino	**1.55** [1.44, 1.67]	**1.26** [1.15, 1.39]	**1.21** [1.10, 1.33]	1.01 [0.94, 1.08]	1.10 [0.96, 1.26]	**0.73** [0.68, 0.79]
Japanese	**0.78** [0.68, 0.90]	**0.64** [0.53, 0.77]	**0.71** [0.61, 0.84]	**0.64** [0.57, 0.72]	**0.61** [0.49, 0.76]	**0.74** [0.66, 0.81]
Korean	0.88 [0.77, 1.02]	0.89 [0.73, 1.08]	**0.74** [0.61, 0.91]	**0.55** [0.48, 0.63]	**0.49** [0.37, 0.65]	**0.40** [0.35, 0.47]
Asian Indian	**1.42** [1.30, 1.55]	**1.29 [1.16, 1.44]**	**0.86** [0.77, 0.97]	**0.81** [0.76, 0.84]	**0.81** [0.70, 0.95]	**0.45** [0.40, 0.50]

*Note.* Reference group is non-Hispanic White. Bold font indicates statistical significance at *p* < .05 from logistic regression models.

**Table 4.** Women Aged 65 and Older: Population Prevalence and Risk of Disability by Race/Ethnicity.

Race/ethnicity	Independent living	Self-care difficulty	Cognitive difficulty	Ambulatory difficulty	Vision difficulty	Hearing difficulty
Population prevalence						
	Percent [95% CI]	Percent [95% CI]	Percent [95% CI]	Percent [95% CI]	Percent [95% CI]	Percent [95% CI]
White	14.5 [14.4, 14.6]	7.2 [7.12, 7.29]	7.6 [7.53, 7.71]	21.8 [21.7, 22.0]	5.83 [5.76, 5.89]	11.1 [11.0, 11.2]
Vietnamese	**23.4** [21.9, 25.0]	**12.4** [11.3, 13.5]	**14.1** [12.8, 15.4]	**24.7** [23.2, 26.2]	**6.73** [5.84, 7.61]	**10.4** [9.49, 11.4]
Chinese	**18.6** [17.9, 19.3]	**9.4** [8.84, 9.87]	**8.6** [8.10, 9.07]	**18.6** [17.9, 19.3]	**5.11** [4.65, 5.57]	**8.2** [7.74, 8.75]
Filipino	**18.5** [17.7, 19.3]	**8.1** [7.49, 8.72]	**9.2** [8.51, 9.80]	22.0 [21.2, 22.9]	**5.11** [4.64, 5.59]	**9.5** [8.83, 10.1]
Japanese	**17.7** [16.5, 18.9]	**8.1** [7.33, 8.88]	**10.6** [9.80, 11.5]	**19.1** [18.0, 20.3]	**4.63** [4.03, 5.23]	11.3 [10.4, 12.1]
Korean	14.1 [13.0, 15.3]	7.1 [6.14, 8.04]	7.6 [6.80, 8.47]	**16.7** [15.4, 18.0]	**4.50** [3.80, 5.19]	**7.8** [6.89, 8.63]
Indian	**19.0** [17.9, 20.2]	**8.9** [8.05, 9.66]	**7.5** [6.88, 8.07]	22.5 [21.3, 23.6]	**4.78** [4.36, 5.21]	**8.3** [7.54, 9.07]
Unadjusted Odds Ratios (O.R.)						
	O.R. [95% CI]	O.R. [95% CI]	O.R. [95% CI]	O.R. [95% CI]	O.R. [95% CI]	O.R. [95% CI]
Vietnamese	**1.81** [1.66, 1.97]	**1.82** [1.65, 2.01]	**1.99** [1.79, 2.21]	**1.18** [1.08, 1.28]	**1.17** [1.01, 1.34]	0.93 [0.84, 1.03]
Chinese	**1.35** [1.28, 1.42]	**1.33** [1.25, 1.42]	**1.14** [1.07, 1.21]	**0.82** [0.78, 0.86]	**0.87** [0.79, 0.96]	**0.72** [0.67, 0.77]
Filipino	**1.34** [1.27, 1.42]	**1.14** [1.05, 1.24]	**1.22** [1.13, 1.32]	1.01 [0.96, 1.06]	**0.87** [0.79, 0.96]	**0.84** [0.78, 0.90]
Japanese	**1.27** [1.17, 1.38]	**1.14** [1.03, 1.26]	**1.44** [1.32, 1.57]	**0.85** [0.79, 0.92]	**0.79** [0.69, 0.90]	1.01 [0.93, 1.10]
Korean	0.97 [0.89, 1.07]	0.98 [0.85, 1.14]	1.00 [0.89, 1.13]	**0.72** [0.65, 0.79]	**0.76** [0.65, 0.90]	**0.67** [0.60, 0.76]
Asian Indian	**1.39** [1.29, 1.50]	**1.25** [1.13, 1.38]	0.98 [0.90, 1.07]	1.04 [0.97, 1.11]	**0.81** [0.74, 0.89]	**0.72** [0.66, 0.80]
Age-adjusted Odds Ratios (O.R.)						
	O.R. [95% CI]	O.R. [95% CI]	O.R. [95% CI]	O.R. [95% CI]	O.R. [95% CI]	O.R. [95% CI]
Vietnamese	**2.45** [2.23, 2.69]	**2.30** [2.07, 2.56]	**2.42** [2.17, 2.71]	**1.36** [1.25, 1.48]	**1.33** [1.16, 1.55]	1.10 [0.99, 1.22]
Chinese	**1.46** [1.39, 1.54]	**1.39** [1.30, 1.48]	**1.17** [1.10, 1.25]	**0.82** [0.78, 0.87]	**0.88** [0.80, 0.97]	**0.71** [0.66, 0.77]
Filipino	**1.63** [1.54, 1.73]	**1.31** [1.21, 1.42]	**1.38** [1.28, 1.50]	**1.11** [1.06, 1.17]	0.96 [0.87, 1.06]	0.94 [0.87, 1.01]
Japanese	0.86 [0.79, 0.94]	**0.80** [0.72, 0.87]	1.08 [0.99, 1.18]	**0.63** [0.58, 0.68]	**0.10** [0.52, 0.69]	**0.71** [0.65, 0.82]
Korean	1.09 [0.98, 1.21]	1.10 [0.95, 1.27]	1.10 [0.98, 1.23]	**0.75** [0.68, 0.83]	**0.82** [0.70, 0.96]	**0.72** [0.64, 0.82]
Asian Indian	**1.90** [1.77, 2.05]	**1.63** [1.48, 1.78]	**1.20** [1.10, 1.31]	**1.23** [1.16, 1.31]	0.96 [0.87, 1.06]	**0.88** [0.80, 0.99]

*Note.* Reference group is non-Hispanic White. Bold font indicates statistical significance at *p* < .05 from logistic regression models.

Age-adjusted odds ratios reflect a similar pattern by gender, with women reporting more disability than men with respect to independent living, self-care, cognitive function, as well as ambulatory function. Vietnamese men had a 32% lower risk of hearing difficulty than non-Hispanic White men, whereas Vietnamese women had a 10% higher risk than non-Hispanic White women, although it was not statistically significant. Similar patterns by gender are evident in the other Asian-origin groups.

## Discussion

We presented updated estimates of the prevalence of disabilities in the six major Asian-origin subgroups and compared them with non-Hispanic Whites. We paid special attention to the Vietnamese-origin older adult population, who were the most disabled group in previous analyses. Using the 2003–2007 ACS data, Yang et al. (2012) found that Vietnamese aged 55 years and over had a higher prevalence of limitations in instrumental activities of daily living (IADL), basic activities of daily living (ADL), as well as cognition, but lower prevalence for physical function and similar prevalence for sensory deficits compared to non-Hispanic Whites. The ADL, IADL, and physical function measures are conceptually equivalent to self-care, independent living, and ambulatory variables in our study. Similarly, using the 2006 ACS data, Fuller-Thomson et al. (2011) found a higher prevalence of disabilities among community-dwelling Vietnamese aged 55 years and older in ADL and cognition, but not functional (ambulatory) limitations or blindness/deafness. Going back further with the 2000 U.S. Census data, Mutchler et al. (2007) found significantly higher reporting of difficulty going outside alone (i.e., independent living) and difficulty performing self-care tasks, but not limitation of physical activities (i.e., ambulatory), by Vietnamese compared to non-Hispanic Whites in adults aged 65 and over.

The results from our analysis are not exactly comparable to previous studies due to the difference in the sample inclusion criteria and changes in question wording. In addition, in 2008, the ACS modified the survey questions related to disability assessment. The wording changes left out the qualification that the impairment must be a long-lasting condition with at least 6 months or more duration. Hearing and vision impairments became two separate questions, with a clarification that vision that can be fixed with wearing glasses is not considered an impairment. Several words (i.e., reaching, lifting, or carrying from physical limitations) or phrases (i.e., getting around inside the home from the ADL question) were deleted or replaced by different words (i.e., “learning” with “making decisions” in cognitive function). Due to these changes, the ACS relabeled these items as assessing difficulties instead of disabilities. Regardless, the items are sufficiently equivalent to show that our study results corroborate with findings from these earlier studies.

We also found differences in disabilities among the Asian-origin groups with the Vietnamese having the worst risk profile, while Japanese and Koreans had the best and even better risk profiles than Whites. Chinese, Filipino, and Asian Indian-origin older adults reported increased risk for independent living, self-care, and cognitive difficulties compared to non-Hispanic Whites, but enjoyed lower risk in vision and hearing difficulties. We also found that women had higher prevalence than men in all areas of difficulty except for hearing. The magnitude of the risk was larger among women than men. Vietnamese women were 2.3 to 2.5 times as likely to have a disability in the area of independent living, self-care, or cognition than non-Hispanic White women, while men were 1.7 to 1.9 times as likely to report difficulties than non-Hispanic White men. However, overall, our updated data showed that the Vietnamese again were the most vulnerable population, having the highest prevalence of disabilities across all areas except hearing, which indicated that they seemed to continue the same patterns of disabilities from over a decade ago.

A number of factors are associated with the higher prevalence of disabilities among Vietnamese older adults. Education level and economic status are two major known factors that play a role in the health of individuals (World Health Organization, 2024). Our data showed that Vietnamese older adults had the highest prevalence of no formal schooling (17.6%) and the highest prevalence of living at or below 150% of the poverty level (13.5%) among all racial/ethnic groups examined. Age of entry (age at immigration) is another factor that is known to influence health (Beck et al., 2012). A sizable number of Vietnamese older adults, 6.3%, entered the U.S. as older adults aged 65 years or older. They spent their entire younger working years in Vietnam and immigrated during their retirement. Thus, they are most likely to have the hardest time adjusting to the U.S. lifestyle and culture, as well as navigating the U.S. healthcare system, given their low English proficiency (Lee et al., 2010). More importantly, many Vietnamese-origin older adults entered the United States as refugees, which has been associated with poorer health in many studies (Markides & Rote, 2019).

This study has several limitations. First, the ACS does not interview everyone in the household but instead relies on one person to complete the survey for everyone in the household, which may introduce biases. Second, the assessments of disability were shortened in 2008 and conceptually changed from disability assessment to difficulty assessment. The shortened version, which has not been validated, could affect the validity of the measures. This study, however, has several key strengths. First, the sample size is sufficiently large to generate stable estimates. Second, the study uses a nationally representative sample of persons living in independent households in the community, for which the findings can be generalized to the U.S. population. Third, we continued using the disaggregated and updated data to examine the variability of Asian subgroups.

## Conclusion

Vietnamese-origin older adults continued to have the highest disability profile compared to non-Hispanic Whites and other major Asian-origin subethnic groups. Some factors associated with the increased disability risk for Vietnamese may include low education level, higher poverty rate, and lack of English fluency. Additional factors such as their pre- and post-migration adversity and predominantly refugee or late-life migration status also need to be considered when examining their disability level and overall health profile. The Vietnamese older adult population in the U.S. is expected to continue to grow. Longitudinal studies are needed to understand the disability trends in the health and healthcare needs of this vulnerable segment of the U.S. population.
